# Nomogram for short-term outcome assessment in AChR subtype generalized myasthenia gravis

**DOI:** 10.1186/s12967-021-02961-9

**Published:** 2021-06-30

**Authors:** Rui Zhao, Ying Wang, Xiao Huan, Huahua Zhong, Zhirui Zhou, Jianying Xi, Yuwei Da, Lin Lei, Ting Chang, Zhe Ruan, Lijun Luo, Shengnan Li, Huan Yang, Yi Li, Sushan Luo, Chongbo Zhao

**Affiliations:** 1 Department of Neurology, Huashan Hospital Fudan University, No.12 Middle Wulumuqi Road, Shanghai, 200040 China; 2 Department of Pharmacy, Huashan Hospital Fudan University, Shanghai, China; 3 Radiation Oncology Center, Huashan Hospital, Fudan University, Shanghai, China; 4 Department of Neurology, Xuanwu Hospital, Capital Medical University, Beijing, China; 5 Department of Neurology, Tangdu Hospital, the Fourth Military Medical University, Xi’an, China; 6 Department of Neurology, Wuhan No.1 Hospital, Wuhan, China; 7 Department of Neurology, Xiangya Hospital, Central South University, Changsha, China

**Keywords:** Generalized myasthenia gravis, Anti-acetylcholine receptor antibody, Minimal symptom expression, Nomogram

## Abstract

**Background:**

An accurate prediction for prognosis can help in guiding the therapeutic options and optimizing the trial design for generalized myasthenia gravis (gMG). We aimed to develop and validate a predictive nomogram to assess the short-term outcome in patients with the anti-acetylcholine receptor (AChR) subtype gMG.

**Methods:**

We retrospectively reviewed 165 patients with AChR subtype gMG who were immunotherapy naïve at the first visit from five tertiary centers in China. The short-term clinical outcome is defined as the achievement of minimal symptom expression (MSE) at 12 months. Of them, 120 gMG patients from Huashan Hospital were enrolled to form a derivation cohort (n = 96) and a temporal validation cohort (n = 24) for the nomogram. Then, this nomogram was externally validated using 45 immunotherapy naïve AChR subtype gMG from the other four hospitals. Multivariate logistic regression was used to screen independent factors and construct the nomogram.

**Results:**

MSE was achieved in 70 (72.9%), 20 (83.3%), and 33 (73.3%) patients in the training, temporal validation, and external validation cohort, respectively. The duration ≤ 12 months (*p* = 0.021), ocular score ≤ 2 (*p* = 0.006), QMG score > 13 (*p* = 0.008), and gross motor score ≤ 9 (*p* = 0.006) were statistically associated with MSE in AChR subtype gMG. The nomogram has good performance in predicting MSE as the concordance indexes are 0.81 (95% CI, 0.72–0.90) in the development cohort, 0.944 (95% CI, 0.83–1.00) in the temporal validation cohort, and 0.773 (95% CI, 0.63–0.92) in the external validation cohort.

**Conclusion:**

The nomogram achieved an optimal prediction of MSE in AChR subtype gMG patients using the baseline clinical characters.

**Supplementary Information:**

The online version contains supplementary material available at 10.1186/s12967-021-02961-9.

## Background

Myasthenia gravis (MG) is an autoimmune disorder characterized by pathological autoantibody-mediated transmission defect in neuromuscular junctions (NMJ) of ocular, bulbar, limb, respiratory, and axial muscles. It can be further divided into different subgroups according to the presentations, antibody specificity, and onset age due to the clinical heterogeneity [1]. Based on the involved muscle, there are approximately 80% of patients develop generalized weakness [2]. Of these generalized myasthenia gravis (gMG) patients, 85% are seropositive for anti-acetylcholine receptor (AChR) antibodies [3]. Therefore, anti-AChR antibody-positive gMG patients account for the majority of MG and also are the main participants in the clinical trials for new immunotherapies.

Therapeutic response and the outcome for gMG patients are critical concerns in clinical practice. Conventional immunotherapies for gMG include corticosteroids and immunomodulatory agents (e.g., azathioprine, mycophenolate mofetil, methotrexate, cyclosporine, and tacrolimus) [4]. A predictive model has been developed for evaluating the corticosteroid-induced initial worsening in a prospective cohort [5]. However, there is still an unmet need for developing a model to predict the clinical outcome for gMG patients [6], especially in the era with emerging therapies development such as eculizumab and neonatal Fc receptor inhibitors [7–9]. For the MG patients who are likely to achieve remission, the benefit from the excessive treatment maybe not be cost-effective [10]. Longitudinal studies provided evidence that approximately 75% of MG patients had an optimal outcome with remission, confined ocular involvement, or mild weakness, while only 7% achieved complete stable remission within a decade [1]. Recently, minimal symptom expression (MSE) that is defined as the patient-reported MG activity of daily living (MG-ADL) scale 0–1 has been used to evaluate the clinical efficacy of efgartigimod in gMG [11]. In comparison to other measures, MSE may provide a more representative outcome measurement for the majority of gMG patients.

In this study, we aim to develop and validate a nomogram for predicting the clinical short-term outcome for gMG patients using the baseline clinical characteristics.

## Methods

### Study design and patient recruitment

There are 1193 MG patients registered in a tertiary referral diagnostic center in Huashan Hospital from August 8, 2012, through December 18, 2020. The inclusion criteria were (1) onset symptoms and signs compatible with gMG; (2) immunotherapy naive at baseline; (3) seropositive for anti-AChR antibody; (4) MG-ADL score > 1 at baseline; (5) follow-up period longer than half a year from baseline; (6) exclusion of other MG mimicking diseases including Lambert–Eaton myasthenic syndrome, peripheral neuropathy, myopathies, and motor neuron diseases. Eligible patients with the integrated baseline data recruited from February 13, 2017, through August 2, 2019, were included in the training cohort for the development of the nomogram, and those recruited from August 2, 2019, through March 13, 2020, were included into the temporal validation cohort. Then, the nomogram was externally validated using 45 anti-AChR antibody-positive gMG patients who have not received immunotherapy from May 2015 to May 2021 at 4 tertiary centers in China (Xiangya Hospital, Xuanwu Hospital, Tangdu Hospital, and Wuhan No.1 Hospital).

The clinical baseline variables include gender, age at onset, the comorbidities of autoimmune disease, and disease duration. The age at onset of MG is classified into three subgroups including early-onset (10–49 years), late-onset (50–64 years), and elderly-onset (65 years or older) [12]. The concurrent autoimmune diseases identified in our cohort include Graves' disease, Hashimoto's autoimmune thyroiditis, type 1 diabetes mellitus, immune thrombocytopenic purpura, autoimmune hemolytic anemia, and vitiligo [13]. The disease duration is defined as the period from the onset of weakness symptoms of MG to the first visit to our hospital. The MG associated clinical features include Myasthenia Gravis Foundation of America (MGFA) classification, thymoma concurrence, history of thymectomy, MG worsening, anti-AChR antibodies titers, pyridostigmine dosage, manual muscle test (MMT) score, MG-ADL score, and the related subscores (bulbar, respiratory, ocular, and limb score), and quantitative myasthenia gravis (QMG) score and the related subscores (extraocular muscle, bulbar muscle, gross motor, and axial motor score). The presence of thymoma is determined by a computed tomography scan. MG worsening is defined as a substantial exacerbation in muscle weakness and fatigability, or increased medication [14]. The anti-AChR antibodies titer was measured by enzyme-linked immunosorbent assay (ELISA, Euroimmun, Lübeck, Germany) and the cut-off value was 0.50 nmol/L.

We divided the total MG-ADL score into four subscores: (1) Ocular score: double vision and eyelid droop; (2) Bulbar score: talking, chewing, and swallowing activities; (3) Respiratory score: the activity of breathing; (4) Limb score: the ability to brush teeth or comb hair, and arise from a chair. For QMG score, it was divided into 5 subscores: (1) Extraocular muscle score: first three items (double vision on lateral gaze, ptosis, and facial muscles); (2) Bulbar muscle: score fourth and fifth items (swallowing 4 oz. water, and the onset of dysarthria); (3) Gross motor score: sixth, seventh, ninth, and tenth, twelfth, and thirteenth items (arms outstretched, hands grip, and legs outstretched); (4) Axial motor score: eleventh item (head lifted); (5) Respiratory score: eighth item (Vital capacity, % predicted).

### Statistical analysis

Our analysis showed that the continuous demographic characteristics data in this study were not normally distributed. The missing data of thymoma, thymectomy, anti-AChR Abs titer, and pyridostigmine dosage account for less than 10%. These missing data were missed at random and replaced by the average of the observed values. Continuous variables were expressed as medians (quartiles) and compared between groups using the Mann–Whitney *U* test. Categorical variables were expressed as frequencies (percentages) and were tested using the *χ2* test or Fisher exact test. To determine the cut-off values of the continuous variable, we created receiver operating characteristic curves for “MSE” and defined them as the points on the ROC curve where Youden's index reached the highest. The significance of each variable in the training cohort was analyzed using univariate and multivariate logistic regression analyses. Variables showing statistical (*P* < 0.1) and clinical significance of the univariate analysis were included in the multivariate logistic regression analysis to develop the nomogram to predict whether a patient would achieve MSE.

The variance inflation factors (VIFs) were generated to examine individual predictors for potentially strong contributions to multicollinearity. The discrimination performance of this nomogram was measured by the concordance index (C-index) in the training and validation cohorts. The Hosmer–Lemeshow test was applied to assess the agreement between nomogram predicted and observed probabilities. All analyses were performed using IBM SPSS version 20.0 (SPSS Company, Chicago, IL, USA) and R software (R version 4.0.3, USA).

## Results

### Patient demographic characteristics

**Fig. 1:**
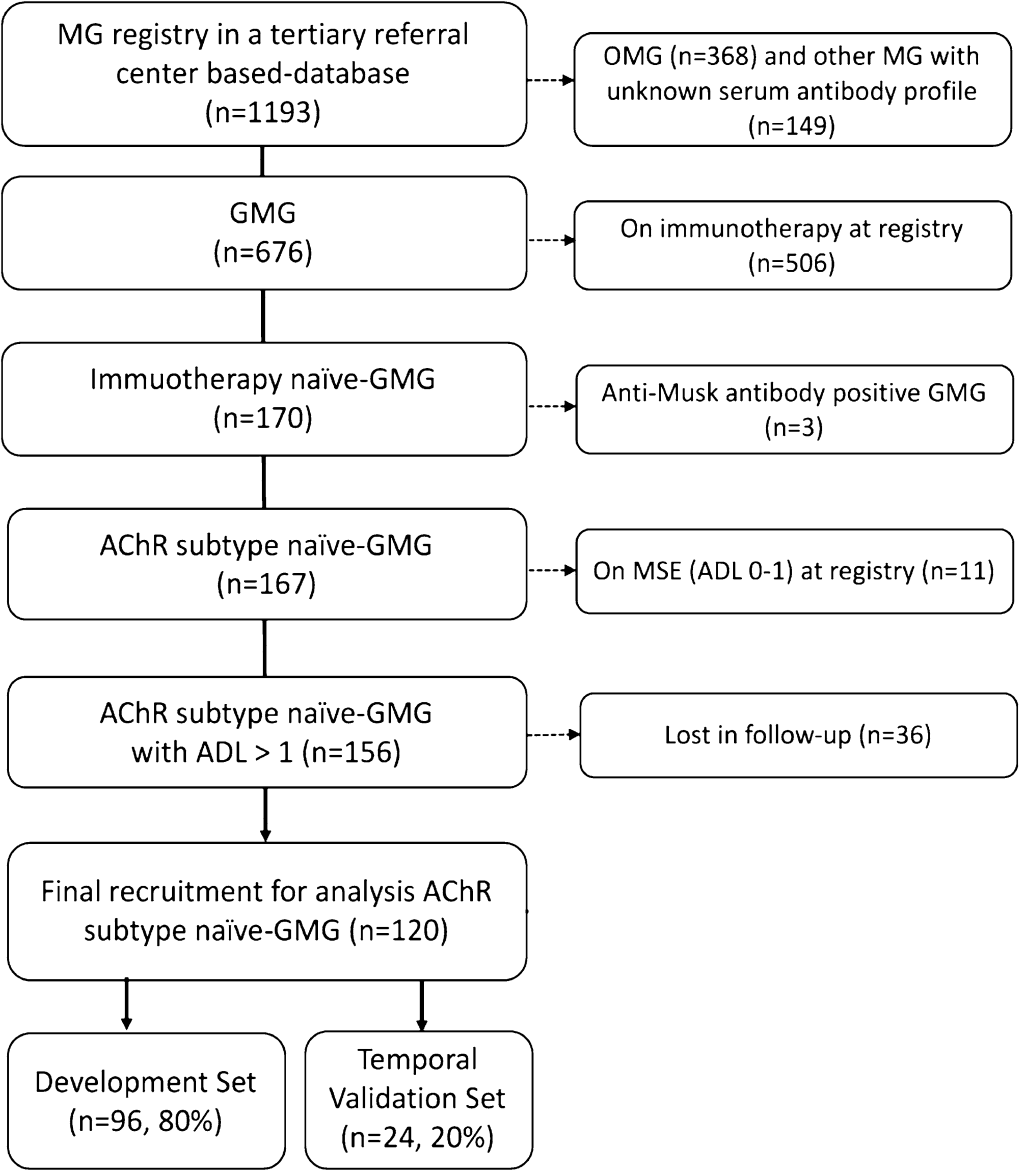
Flow diagram of the enrollment of study participants and the classification of training and temporal validation set

A total of 1193 MG patients have been initially registered in our referral center-based database. According to the inclusion flowchart, we finally enrolled 120 AChR subtype gMG patients with no immunotherapies at the baseline registry (Fig. 1). Of these gMG patients, 96 and 24 patients were then included in the training and temporal validation cohort splitting by time. Besides, 45 AChR subtype gMG patients from the other 4 centers were enrolled for external validation.

The baseline clinical characteristics and the outcome of MG patients in the training and temporal validation cohort were comparable, except for the differences in the respiratory score (*p* = 0.035), and respiratory muscle score (*p* = 0.002) (Table 1). In the development set, 70 patients (72.9%) achieved MSE and the median disease duration was 7 (3.0–30.5) months. At baseline, 34% of patients were diagnosed to have concurrent thymoma and 24% had undergone thymectomy in the development set. The ADL and QMG scores of the development cohort were 5 (4.0–8.0) and 11 (9.0–14.0), respectively.

**Table 1:** The Baseline Demographic Characteristics and Outcome of the Development and Validation Set

Variables	Subgroups	Development set (n = 96) No. of patients (%) Median (range)	Temporal validation set (n = 24) No. of patients (%) Median (range)	*P* value
Outcome	Non MSE	26 (27.1)	4 (16.7)	0.292
	MSE	70 (72.9)	20 (83.3)	
Gender	Male	46 (47.9)	15 (62.5)	0.201
	Female	50 (52.1)	9 (37.5)	
Age at onset	EOMG	62 (64.6)	16 (66.7)	0.815
	LOMG	25 (26.0)	5 (20.8)	
	Elderly-onset MG	9 (9.6)	3 (12.5)	
MGFA classification	II	62 (64.6)	16 (66.7)	0.999
	III	29 (30.2)	7 (29.2)	
	IV	5 (5.2)	1 (4.2)	
Thymoma	No	62 (66.0)	19 (79.2)	0.213
	Yes	32 (34.0)	5 (20.8)	0.633
Thymectomy	No	70 (74.5)	19 (79.2)	
	Yes	24 (25.5)	5 (20.8)	
Worsening	No	24 (25.0)	9 (37.5)	0.22
	Yes	72 (75.0)	15 (62.5)	
Autoimmune disease	No	86 (89.6)	20 (83.3)	0.619
	Yes	10 (10.4)	4 (16.7)	
Disease duration, months		7 (3.0–30.5)	4 (2.0–14.5)	0.305
Anti-AChR Abs titer, nmol/L		6 (2.6–10.3)	8 (2.6–12.4)	0.316
Pyridostigmine dosage, mg/day		180 (90.0–180.0)	180 (180.0–180.0)	0.557
MMT score		14 (7.0–19.0)	14 (7.3–19.8)	0.743
MG-ADL score		5 (4.0–8.0)	5 (4.0–7.0)	0.377
Bulbar score		2 (1.0–3.0)	1 (0–2.8)	0.145
Respiratory score		0 (0–1.0)	0 (0–0)	0.035*
Limb score		1 (0–2.0)	0 (0–1.0)	0.103
Ocular score		3 (1.0–4.0)	3 (2.0–4.0)	0.182
QMG score		11 (9.0–14.0)	12 (7.0–16.0)	0.638
Extraocular muscle score		3 (1.0–4.0)	4 (2.0–5.0)	0.038
Bulbar muscle score		0 (0–1.0)	1 (0–2.0)	0.110
Respiratory muscle score		0 (0–1.0)	0 (0–0)	0.002*
Gross motor score		6 (4.0–8.0)	6 (2.5–8.0)	0.220
Axial motor score		1 (1.0–2.0)	1 (1.0–2.0)	0.869

*MSE* minimal symptom expression, *EOMG* early-onset myasthenia gravis, *LOMG* late-onset myasthenia gravis, *MG* myasthenia gravis, *MGFA* Myasthenia Gravis Foundation of America, *AChR* acetylcholine receptor, *Abs* antibodies, *MMT* manual muscle test, *MG-ADL* myasthenia gravis-activity of daily living, *QMG* quantitative myasthenia gravis

*Statistical significance (*α* = 0.05)

The clinical characteristics of the external validation and development groups are summarized in Table 2. The frequency of MSE was similar for the development (72.9%) and external validation groups (73.3%), whereas there were some differences between these groups regarding the frequency of thymectomy, disease duration, anti-AChR Abs titer, MMT score, bulbar score, bulbar muscle score, and gross motor score.

**Table 2:** The Baseline Demographic Characteristics and Outcome of the Development and External Validation Set

Variables	Subgroups	Development set (n = 96) No. of patients (%) Median (range)	External validation set (n = 45) No. of patients (%) Median (range)	*P* value
Outcome	Non MSE	26 (27.1)	12 (26.7)	0.959
	MSE	70 (72.9)	33 (73.3)	
Gender	Male	46 (47.9)	22 (48.9)	0.914
	Female	50 (52.1)	23 (51.1)	
Age at onset	EOMG	62 (64.6)	23 (51.1)	0.096
	LOMG	25 (26.0)	12 (26.7)	
	Elderly-onset MG	9 (9.6)	10 (22.2)	
MGFA classification	II	62 (64.6)	21 (46.7)	0.128
	III	29 (30.2)	20 (44.4)	
	IV	5 (5.2)	4 (8.9)	
Thymoma	No	62 (66.0)	32 (71.1)	0.543
	Yes	32 (34.0)	13 (28.9)	
Thymectomy	No	70 (74.5)	41 (91.1)	0.022^*^
	Yes	24 (25.5)	4 (8.9)	
Worsening	No	24 (25.0)	7 (15.6)	0.207
	Yes	72 (75.0)	38 (84.4)	
Autoimmune disease	No	86 (89.6)	37 (82.2)	0.222
	Yes	10 (10.4)	8 (17.8)	
Disease duration, months		7 (3.0–30.5)	2 (1.0–6.0)	0.001*
Anti-AChR Abs titer, nmol/L		6 (2.6–10.3)	8 (4.5–20.1)	0.018*
Pyridostigmine dosage, mg/day		180 (90.0–180.0)	180 (0–210.0)	0.528
MMT score		14 (7.0–19.0)	50 (41.5–50)^#^	0.001*
MG-ADL score		5 (4.0–8.0)	6 (4.0–9.5)	0.151
Bulbar score		2 (1.0–3.0)	2 (1.0–4.0)	0.026*
Respiratory score		0 (0–1.0)	0 (0–1.0)	0.540
Limb score		1 (0–2.0)	0 (0–2.0)	0.651
Ocular score		3 (1.0–4.0)	3 (2.0–4.0)	0.458
QMG score		11 (9.0–14.0)	10 (7.0–16.5)	0.485
Extraocular muscle score		3 (1.0–4.0)	3 (2.5–4.5)	0.068
Bulbar muscle score		0 (0–1.0)	1 (0–3.0)	0.001*
Respiratory muscle score		0 (0–1.0)	0 (0–1.0)	0.357
Gross motor score		6 (4.0–8.0)	5 (2.0–8.5)	0.042*
Axial motor score		1 (1.0–2.0)	1 (0–2.0)	0.097

*MSE* minimal symptom expression, *EOMG* early-onset myasthenia gravis, *LOMG* late-onset myasthenia gravis, *MG* myasthenia gravis, *MGFA* Myasthenia Gravis Foundation of America, *AChR* acetylcholine receptor, *Abs* antibodies, *MMT* manual muscle test, *MG-ADL* myasthenia gravis-activity of daily living, *QMG* quantitative myasthenia gravis

^*^Statistical significance (*α* = 0.05)

^#^There are only Wuhan No.1 Hospital record the MMT score (n = 9)

### Short-term clinical outcome assessment

MSE status was achieved in 70 (72.9%), 20 (83.3%), and 33 (73.3%) patients in the training, temporal validation, and external validation cohorts at 12 months after baseline recruitment. For the patients who did not achieve MSE, the median ADL scores were 3 (range 3–6), 4.5 (range 3.25–5), and 2.5 (range 2–7) in the training, temporal validation, and external validation cohorts, respectively.

In the training and temporal validation groups in Huashan Hospital, the initial dose and dose-escalating manner of prednisone depended on the physician's decision. The final oral prednisone dose for each patient was at 0.8 mg to 1 mg/kg and azathioprine, tacrolimus, or mycophenolate mofetil as immunosuppressants concurrent with oral prednisone. Three patients had received rescue therapies including immunoglobulin and plasma exchange.

### Nomogram development and validation

**Fig. 2:**
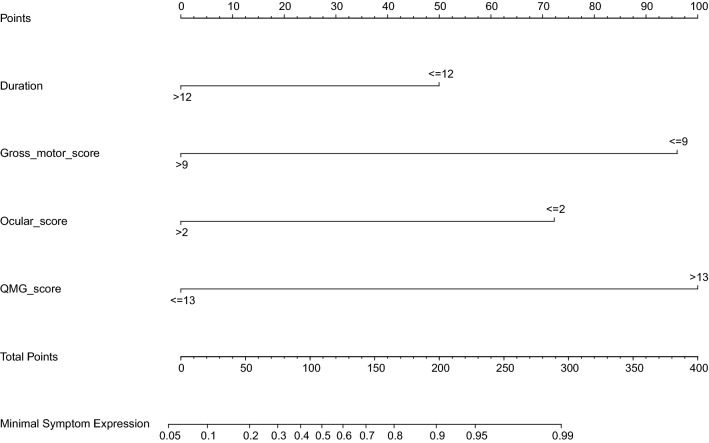
Nomogram to estimate the probability of MSE in immunotherapy naïve gMG patients with anti-AChR antibodies. To use this nomogram, find the position of each variable on the corresponding axis, draw a line to the points axis for the number of points, add the points from all of the variables, and draw a line from the total points axis to determine the MSE probabilities at the lower line of the nomogram

We identified three risk factors significantly associated with MSE including duration, ocular score, and gross motor score (*p* < 0.1) (Table 3). Considering the clinical significance, we also included the QMG score (*p* = 0.155) along with these statistically significant variables into the multivariate logistic regression. All these above variables were independently associated with MSE (*p* < 0.05), with results reported as odds ratio (95% CI), duration ≤ 12 months (3.45 [1.23–10.24]), ocular score ≤ 2 (6.00 [1.82 to 24.58]), QMG score > 13 (11.95 [2.31 to 95.82]), and gross motor score ≤ 9 (10.82 [2.22–69.13]). The VIFs of them were 1.01, 1.25, 1.78, and 1.84 respectively, suggesting that there was no multiple collinearity among the four independent risk factors. We then used these four factors to establish an individualized prediction nomogram, which can calculate the total point for each gMG patient with anti-AChR antibodies and converted it to predicted probabilities of MSE (Fig. 2). This nomogram was then validated in both the temporal validation cohort derived from Huashan Hospital and the external validation cohort.

**Table 3:** Univariate and multivariate logistic regression models for minimal symptom expression in the development group

Variables	Subgroups	Univariate analysis			Multivariate analysis**		
		OR	95% CI	*P* value	OR	95% CI	*P* value
Gender	Male	1					
	Female	0.5	0.19–1.25	0.146			
Age at onset	EOMG	1					
	LOMG	1.2	0.42–3.73	0.744			
	Elderly-onset MG	0.76	0.18–3.90	0.713			
MGFA classification	II	1					
	III	1.19	0.44–3.45	0.741			
	IV	0.57	0.09–4.58	0.553			
Thymoma	Yes	1					
	No	0.71	0.25–1.86	0.494			
Thymectomy	No	1					
	Yes	2.2	0.73–8.23	0.192			
Worsening	Yes						
	No	2.36	0.78–8.80	0.155			
Autoimmune disease	No	1					
	Yes	0.85	0.22–4.21	0.827			
MMT score	> 26	1					
	≤ 26	2.36	0.54–9.72	0.229			
Anti-AChR Abs titer, nmol/L	> 9	1					
	≤ 9	0.49	0.15–1.37	0.197			
Duration, months	> 12	1			1		
	≤ 12	4.41	1.73–11.90	0.002^**^	3.45	1.23–10.24	0.021
Pyridostigmine dosage, mg/day	≤ 240						
	> 240	0.46	0.10–2.50	0.339			
MG-ADL score	> 3	1					
	≤ 3	2.74	0.69–18.36	0.207			
Bulbar score	≤ 1	1					
	> 1	2.01	0.81–5.19	0.136			
Respiratory function	Normal	1					
	Abnormal	1.53	0.56–4.65	0.426			
Limb score	≤ 1	1					
	> 1	1.51	0.57–4.31	0.418			
Ocular score	> 2				1		
	≤ 2	2.87	1.11–8.15	0.036^**^	6.00	1.82–24.58	0.006
QMG score	≤ 13	1			1		
	> 13	2.36	0.78–8.80	0.155	11.95	2.31–95.82	0.008
Extraocular muscle score	≥ 1	1					
	0	2.52	0.84–9.39	0.124			
Bulbar muscle score	≥ 1	1					
	0	0.51	0.17–1.37	0.198			
Respiratory muscle score	≥ 80	1					
	65–79	0.98	0.36–2.79				
	50–64	0.75	0.28–9.48				
	< 50	0.81	0.07–16.83				
Gross motor score	> 9	1			1		
	≤ 9	2.67	0.90–7.81	0.072^*^	10.82	2.22–69.13	0.006
Axial motor score	> 2	1					
	≤ 2	1.70	0.33–7.48	0.493			

*CI,* confidence interval, *OR* odds ratio, *MSE* minimal symptom expression, *EOMG* early-onset myasthenia gravis, *LOMG* late-onset myasthenia gravis, *MG* myasthenia gravis, *MGFA* Myasthenia Gravis Foundation of America, *AChR* acetylcholine receptor, *Abs* antibodies, *MMT* manual muscle test, *MG-ADL* myasthenia gravis-activity of daily living, *QMG* quantitative myasthenia gravis

*Statistical significance (*α* = 0.1)

**Statistical significance (*α* = 0.05)

**Fig. 3:**
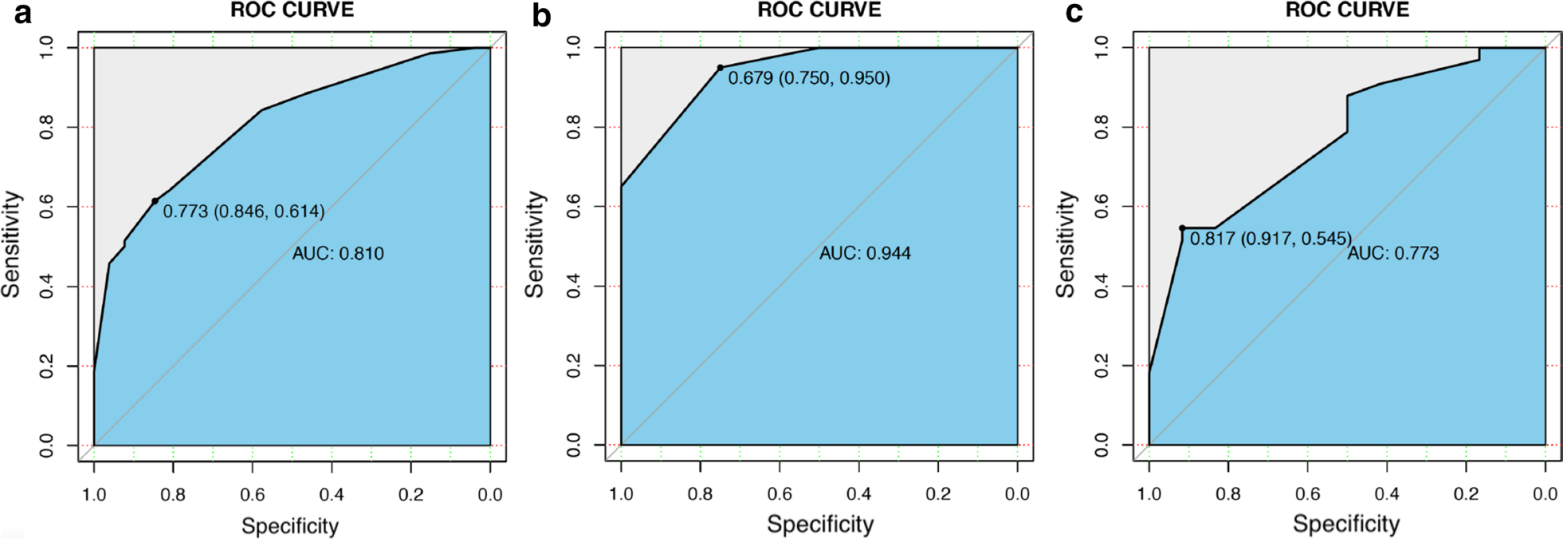
The ROC curves represent the discrimination ability of the model measured by the C-index are 0.810 (95% CI, 0.72–0.90), 0.944 (95% CI 0.83–1.00), and 0.773 (95% CI 0.63–0.92) in the development (**a**), temporal validation cohort (**b**), and external validation cohort (**c**), respectively

The ability of this nomogram to differentiate between patients who do or do not achieve MSE is excellent as the C-indexes are 0.810 (95% CI, 0.72–0.90), 0.944 (95% CI 0.83–1.00), and 0.773 (95% CI, 0.63–0.92) in the development, temporal validation, and external validation cohorts, respectively (Fig. 3). Besides, the p-values of the Hosmer–Lemeshow test are 0.98, 0.99, and 0.61 for the development, temporal validation, and external validation sets, which indicates good agreement between nomogram predicted and observed probabilities.

## Discussion

To optimize the clinical management, the need to identify the patients with good prognoses using the baseline characteristics is unmet. In this multicenter study, we suggest that disease duration ≤ 12 months, ocular score ≤ 2, QMG score > 13, and gross motor score ≤ 9 before immunosuppressants administration are significant predictors for reaching the status of MSE in AChR subtype gMG patients.

For patients with anti-AChR antibodies, these antibodies bind and activate the complement cascade at the NMJ, resulting in the postsynaptic folds degeneration of skeletal muscle [12]. It has been shown that chronic anticholinesterase treatment in rats could destroy the postsynaptic membrane [15]. Around 80 patients (83.3%) included in the training cohort have only administered pyridostigmine before the first visit. From our study, we indicated that the patients who had a duration from onset to immunosuppressive therapies shorter than 12 months had a better outcome, which may be due to an alleviate NMJ destruction. A systematic review also identified the duration between onset and diagnosis (< 1 year) was a strong predictor of remission for MG patients [6].

We also found that MG patients whose baseline QMG score more than 13 were more likely to achieve MSE. Previous logistic regression analysis had also confirmed that the high baseline QMG score serves as an independent predictor of response to immunotherapy [16]. As a result, these patients with more severe weakness symptoms measured by the QMG appear to respond better to treatments and have good prognoses.

QMG is a valid subject scale to assess the severity of the weakness. However, the items are linearly scored and not weighted. Based on the clinical practicability, though the signs of poor vital capacity and spontaneous ptosis gain the same QMG score, the degree of disability experienced by the former is more severe. As a result, MGFA also recommended “weighting” specific sub-scores of the QMG [17]. In this study, QMG has been divided into five functional subscores and the gross motor score ≤ 9 was a significant risk factor for MSE in MG. In an observational study of 2000 MG patients over sixty years, David et al. have found some MG patients who experienced remission also had mild weakness of legs or orbicularis oculi [18]. It was consistent with our study that gross motor score and ocular score were associated with MSE.

Our database documented the doses of corticosteroid or immunosuppressive drugs in gMG patients. The combination of immunosuppressive drugs was analyzed using univariate and multivariate logistic regression. However, there were 26 (27%) missing values in the training cohort that were replaced by fifty multiple imputations (MIs) counterparts. The multivariable model of MIs data showed that the combination of immunosuppressive drugs (p = 0.034, OR 0.22 [0.06–0.87]) with the above four risk factors was associated with MSE(Additional file 1: Table S1). Therefore, the baseline clinical characteristics other than advanced immunotherapy were vital predictors for MSE of gMG patients with anti-AChR antibodies. A recent study also revealed that the higher prednisolone dosage and the more frequent plasmapheresis were associated with the treatment-resistant outcome for MG patients [19]. The MIs model had good agreement with the nonimputed model and there was little difference between the ORs for the original and the MIs models. Given the high percentage of missing data, the final analyses were performed on the original model.

With the development of novel therapy for MG, MSE has severed as a patient-reported primary outcome measure in clinical trials. Tough minimal manifestation status (MMS) is the goal for the treatment of MG, MSE is more available in the clinical trials for a long follow-up due to its unique advantages. MSE is not only able to reflect the patient’s experienced disease fluctuations symptom during a long period, but easy to acquire in an online follow-up study with no need of specialized equipment or training [20, 21]. This study showed that the baseline characteristics before starting immunotherapy are determinants for MSE. As a result, we suggest clinical trials that use MSE as an endpoint should pay more attention to the distribution of duration, ocular score, QMG score, and gross motor score in the different groups to decrease selection bias.

There are several limitations of our study. Firstly, the records of therapy were insufficient. However, we used the statistic method including internal temporal validation and MIs to minimize these shortcomings. Secondly, patients who did not have sufficient clinical records were excluded, which may result in selection bias. Finally, the nomogram was based only on Chinese gMG patients. This nomogram may have some restrictions to predict the outcome for gMG patients from others areas due to the different treatment methods. The prospective and large-scale analysis is required to test and verify this nomogram.

## Conclusions

In conclusion, we develop and validate a nomogram to predict the probability to achieve MSE using the baseline clinical characteristics. The prediction would help in clinical decision-making and prognosis monitoring. Simultaneously, we suggest that these baseline clinical characteristics should be evaluated before the selection of participants in MG trials to avoid potential bias.

## Supplementary Information

**Additional file 1. Table S1.** Univariate and multivariate Logistic regression models for minimal symptom expression in the development group.

## Data Availability

The datasets used and/or analysed during the current study are available from the corresponding author on reasonable request.

## Abbreviations

MG: Myasthenia gravis
NMJ: Neuromuscular junctions
gMG: Generalized myasthenia gravis
AChR: Acetylcholine receptor
MSE: Minimal symptom expression
MG-ADL: Myasthenia gravis activities of daily living
MGFA: Myasthenia Gravis Foundation of America
MMT: Manual muscle test
QMG: Quantitative myasthenia gravis
VIFs: Variance inflation factors
C-index: Concordance index
MMS: Minimal manifestation status
CI: Confidence interval
OR: Odds ratio
