# Supplementary material for: Nomogram for short-term outcome assessment in AChR subtype generalized myasthenia gravis
Source: J Transl Med. 2021 Jun 30;19:285. doi: 10.1186/s12967-021-02961-9 (PMC8247112; doi:10.1186/s12967-021-02961-9)
Supplement: Supplementary file 1 — Additional file 1. Table S1. Univariate and multivariate Logistic regression models for minimal symptom expression in the development group. [file 12967_2021_2961_MOESM1_ESM.docx]

Nomogram for short-term outcome assessment in AChR subtype generalized myasthenia gravis

Rui Zhao, Ying Wang, Xiao Huan, Huahua Zhong, Zhirui Zhou, Jianying Xi, Yuwei Da, Lin Lei, Ting Chang, Zhe Ruan, Lijun Luo, Shengnan Li, Huan Yang, Yi Li, Sushan Luo, Chongbo Zhao.

**Correspondence to**: Sushan Luo (luosushan@fudan.edu.cn) and Chongbo Zhao ([zhao_chongbo@fudan.edu.cn](mailto:zhao_chongbo@fudan.edu.cn)), Huashan Hospital Fudan University, No.12 Middle Wulumuqi Road, Shanghai, 200040, China.

Supplemental Table 1. Univariate and multivariate Logistic regression models for minimal symptom expression in the development group.

| Variables | Subgroups | Univariate analysis | | | Multivariate analysis^**^ | | |
| --- | --- | --- | --- | --- | --- | --- | --- |
|  |  | OR | 95% CI | P value | OR | 95% CI | *P* value |
| Gender | Male | 1 |  |  |  |  |  |
|  | Female | 0.5 | 0.19-1.25 | 0.146 |  |  |  |
| Age at onset | EOMG | 1 |  |  |  |  |  |
|  | LOMG | 1.2 | 0.42-3.73 | 0.744 |  |  |  |
|  | Elderly-onset MG | 0.76 | 0.18-3.90 | 0.713 |  |  |  |
| MGFA classification | II | 1 |  |  |  |  |  |
|  | III | 1.19 | 0.44-3.45 | 0.741 |  |  |  |
|  | IV | 0.57 | 0.09-4.58 | 0.553 |  |  |  |
| Thymoma | Yes | 1 |  |  |  |  |  |
|  | No | 0.71 | 0.25-1.86 | 0.494 |  |  |  |
| Thymectomy | No | 1 |  |  |  |  |  |
|  | Yes | 2.2 | 0.73-8.23 | 0.192 |  |  |  |
| Worsening | Yes |  |  |  |  |  |  |
|  | No | 2.36 | 0.78-8.80 | 0.155 |  |  |  |
| Autoimmune disease | No | 1 |  |  |  |  |  |
|  | Yes | 0.85 | 0.22-4.21 | 0.827 |  |  |  |
| MMT score | >26 | 1 |  |  |  |  |  |
|  | <=26 | 2.36 | 0.54-9.72 | 0.229 |  |  |  |
| Anti-AChR Abs titer, nmol/L | >9 | 1 |  |  |  |  |  |
|  | <=9 | 0.49 | 0.15-1.37 | 0.197 |  |  |  |
| Duration, months | >12 | 1 |  |  | 1 |  |  |
|  | <=12 | 4.41 | 1.73-11.90 | 0.002^**^ | 3.71 | 1.20-11.49 | 0.025 |
| Pyridostigmine dosage, mg/day | <=240 |  |  |  |  |  |  |
|  | >240 | 0.46 | 0.10-2.50 | 0.339 |  |  |  |
| MG-ADL score | >3 | 1 |  |  |  |  |  |
|  | <=3 | 2.74 | 0.69-18.36 | 0.207 |  |  |  |
| Bulbar score | <=1 | 1 |  |  |  |  |  |
|  | >1 | 2.01 | 0.81-5.19 | 0.136 |  |  |  |
| Respiratory function | normal | 1 |  |  |  |  |  |
|  | abnormal | 1.53 | 0.56-4.65 | 0.426 |  |  |  |
| Limb score | <=1 | 1 |  |  |  |  |  |
|  | >1 | 1.51 | 0.57-4.31 | 0.418 |  |  |  |
| Ocular score | >2 |  |  |  | 1 |  |  |
|  | <=2 | 2.87 | 1.11-8.15 | 0.036^**^ | 6.59 | 1.64-26.42 | 0.009 |
| QMG score | <=13 | 1 |  |  | 1 |  |  |
|  | >13 | 2.36 | 0.78-8.80 | 0.155 | 16.81 | 2.47-114.35 | 0.005 |
| Extraocular muscle score | >=1 | 1 |  |  |  |  |  |
|  | 0 | 2.52 | 0.84-9.39 | 0.124 |  |  |  |
| Bulbar muscle score | >=1 | 1 |  |  |  |  |  |
|  | 0 | 0.51 | 0.17-1.37 | 0.198 |  |  |  |
| Respiratory muscle score | >=80 | 1 |  |  |  |  |  |
|  | 65-79 | 0.98 | 0.36-2.79 |  |  |  |  |
|  | 50-64 | 0.75 | 0.28-9.48 |  |  |  |  |
|  | <50 | 0.81 | 0.07-16.83 |  |  |  |  |
| Gross motor score | >9 | 1 |  |  | 1 |  |  |
|  | <=9 | 2.67 | 0.90-7.81 | 0.072^*^ | 10.45 | 1.83-59.70 | 0.010 |
| Axial motor score | >2 | 1 |  |  |  |  |  |
|  | <=2 | 1.70 | 0.33-7.48 | 0.493 |  |  |  |
| Combined immunosuppressive regimens | No | 1 |  |  | 1 |  |  |
|  | Yes | 0.32 | 0.11-0.92 | 0.039^**^ | 0.223 | 0.06-0.87 | 0.033 |

Data for Combined immunosuppressive regimens were missing for 26 patients. Therefore, multiple imputations were performed. The Odds Ratios, 95% CI, and P value shown here are from the multiple-imputed model (Chi-squared p=0.01).

CI, confidence interval, OR odds ratio, *MSE* minimal symptom expression, *EOMG* early-onset myasthenia gravis, *LOMG* late-onset myasthenia gravis, *MG* myasthenia gravis, *MGFA* Myasthenia Gravis Foundation of America, *AChR* acetylcholine receptor, *Abs* antibodies, *MMT* manual muscle test, *MG-ADL* myasthenia gravis-activity of daily living, *QMG* quantitative myasthenia gravis

* Statistical significance (α = 0.1)

** Statistical significance (α = 0.05)
